# Evaluation of the Accuracy of Artificial Intelligence (AI) Models in Dermatological Diagnosis and Comparison With Dermatology Specialists

**DOI:** 10.7759/cureus.77067

**Published:** 2025-01-07

**Authors:** Yuto Yamamura, Kazuyasu Fujii, Chisa Nakashima, Atsushi Otsuka

**Affiliations:** 1 Department of Dermatology, Kindai University Hospital, Osakasayama, JPN

**Keywords:** artificial intelligence, clinical evaluation, dermatology, diagnostic accuracy, generative models

## Abstract

Recent advances in generative artificial intelligence (AI) have expanded its applications in diagnostic support within dermatology, but its clinical accuracy requires ongoing evaluation. This study compared the diagnostic performance of three advanced AI models, ChatGPT-4o, Claude 3.5 Sonnet, and Gemini 1.5 Pro, with that of board-certified dermatologists, using a dataset of 30 cases encompassing a variety of dermatological conditions. The AI models demonstrated diagnostic accuracy comparable to, and sometimes exceeding, that of the specialists, particularly in rare and complex cases. Statistical analysis revealed no significant difference in accuracy rates between the AI models and dermatologists, indicating that AI may serve as a valuable supplementary diagnostic tool in dermatological practice. Limitations include a small sample size and potential selection bias. However, these findings underscore the progress in AI’s diagnostic capabilities, supporting further validation with larger datasets and diverse clinical scenarios to confirm its practical utility.

## Introduction

Multimodal generative artificial intelligence (AI) is a technology capable of performing various tasks, such as natural language processing, image generation, and speech synthesis, based on large datasets, and it has garnered attention in the medical field [1]. AI chatbots utilizing large language models are beginning to be applied in diagnostic support, interpretation of test results, and prediction of patient outcomes [2]. The utility of generative AI as a diagnostic tool in the medical field has been reported [3]. In the dermatological field, deep learning research using image data has advanced, suggesting that diagnostic accuracy may rival that of dermatologists [4]. While previous generative AI models have demonstrated utility as diagnostic aids, their diagnostic accuracy has been limited and considered inferior to that of board-certified dermatologists [5,6]. Additionally, the potential for AI models to enhance diagnostic accuracy through the interpretation of dermoscopic images has been highlighted [7]. The aim of this study is to evaluate the diagnostic capabilities of the latest generative AI models and to assess their practical applicability in dermatological practice. In this study, while the diagnostic process of the AI models remains opaque, we aim to evaluate their clinical utility by focusing on the accuracy of diagnostic concordance between AI models and dermatology specialists.

## Materials and methods

Data selection

Thirty cases, including 15 neoplastic and 15 inflammatory dermatological diseases, were randomly selected from the Japanese dermatology journal Hifu no Kagaku. Each case included patient history, clinical findings, clinical photographs, pathological images, and test data.

Evaluation by AI models and dermatologists

The cases were presented to three generative AI models, ChatGPT-4o, Claude 3.5 Sonnet, and Gemini 1.5 Pro, as well as 11 dermatology specialists. The AI models were instructed to provide a diagnosis as a top-class Japanese dermatologist. Diagnoses from the AI models and dermatologists were compared for accuracy. The cases, extracted and edited into an appendix format, included patient history, clinical findings, clinical photographs, pathological images, and test data (see the Appendix for details). The appendix example was extracted from the Japanese dermatology journal Hifu no Kagaku.

Statistical analysis

Diagnostic accuracy rates between the AI models and dermatologists were analyzed using the Mann-Whitney U test. Correlations between the AI models’ and dermatologists’ accuracies were assessed using Spearman’s rank correlation coefficients. Statistical analyses were performed using GraphPad Prism version 9.5.1 (GraphPad Software Inc., Boston, MA), with significance set at p < 0.05.

## Results

Overall diagnostic accuracy

**Figure 1:**
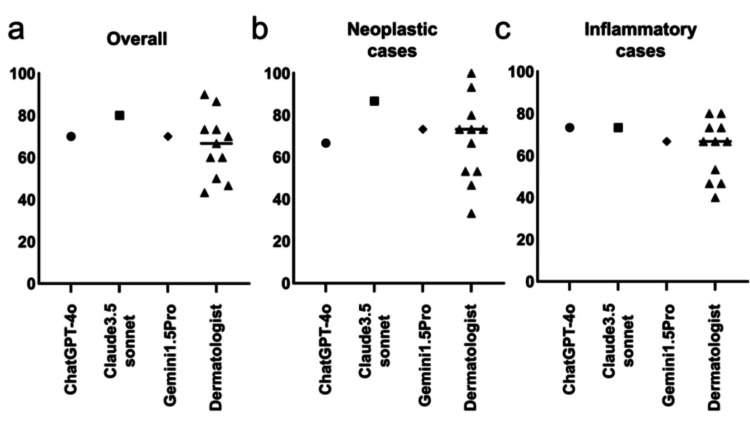
Comparison of diagnostic accuracy rates between AI models and dermatologists for (a) all questions, (b) questions related to neoplastic diseases, and (c) questions related to inflammatory diseases Each plot shows the diagnostic accuracy rate for ChatGPT-4o (●), Claude 3.5 Sonnet (■), Gemini 1.5 Pro (⧫), and 11 dermatologists (▲). The horizontal line represents the average accuracy of the dermatologists AI: artificial intelligence

The overall accuracy rates for the 30 cases were 70% for ChatGPT-4o, 80% for Claude 3.5 Sonnet, 70% for Gemini 1.5 Pro, and an average of 65.4% for the dermatologists (range: 43%-90%; SD: 0.153) (Figure 1a). The Mann-Whitney U test showed no significant differences in accuracy rates between the AI models and dermatologists (U = 11, p = 0.448), with median accuracies of 70.0% for the AI models and 66.67% for the dermatologists (Hodges-Lehmann estimate = -10).

Neoplastic and inflammatory diseases

For neoplastic cases, the accuracy rates were 66.7% for ChatGPT-4o, 86.7% for Claude 3.5 Sonnet, 73.3% for Gemini 1.5 Pro, and an average of 67.9% for the dermatologists (range: 33.3%-100%; SD: 0.200) (Figure 1b). No significant difference was found (U = 13, p = 0.6346), with median accuracies of 73.33% for both groups (Hodges-Lehmann estimate = 0). In inflammatory cases, accuracy rates were 73.3% for both ChatGPT-4o and Claude 3.5 Sonnet, 66.7% for Gemini 1.5 Pro, and 63% for the dermatologists (range: 40%-80%, SD: 0.141) (Figure 1c). Again, no significant difference was found (U = 11.5, p = 0.4725), with median accuracies of 73.33% for the AI models and 66.67% for the dermatologists (Hodges-Lehmann estimate = -6.667).

Correlation analysis

Among cases where dermatologists achieved ≥75% accuracy, ChatGPT-4o and Gemini 1.5 Pro correctly diagnosed 90.9%, while Claude 3.5 Sonnet achieved 100%. For cases with ≤25% dermatologist accuracy, ChatGPT-4o and Claude 3.5 Sonnet achieved 50%, while Gemini 1.5 Pro achieved 25% (Table 1). Weak positive correlations were observed between the accuracy of dermatologists and ChatGPT-4o (ρ = 0.1865, p = 0.3237), with statistically significant correlations found for Claude 3.5 Sonnet (ρ = 0.3788, p = 0.039) and Gemini 1.5 Pro (ρ = 0.4239, p = 0.0196), indicating consistency between the AI models and dermatologists.

**Table 1:** The correct and incorrect diagnoses made by the AI models (ChatGPT-4o, Claude 3.5 Sonnet, and Gemini 1.5 Pro) along with the accuracy rates of the dermatologists. A checkmark (✔) indicates a correct diagnosis, while a cross (×) indicates an incorrect one. The Dermatologists column shows the number of correct diagnoses out of 11 dermatology specialists for each case SCC: squamous cell carcinoma; IgA: immunoglobulin A; AI: artificial intelligence

No.	Diagnosis	ChatGPT-4o	Claude 3.5 sonnet	Gemini 1.5 Pro	Dermatologist
Case 1	Malignant melanoma	✔	✔	✔	11/11
Case 2	Basal cell carcinoma	✔	✔	✔	11/11
Case 3	SCC	✔	✔	✔	9/11
Case 4	Melanocytic nevus	×	×	×	7/11
Case 5	Merkel cell carcinoma	✔	✔	✔	8/11
Case 6	Pagetoid Bowen's disease	×	×	×	8/11
Case 7	Diffuse large B-cell lymphoma	✔	✔	✔	7/11
Case 8	Subcutaneous panniculitis-like T-cell lymphoma	×	✔	✔	8/11
Case 9	Extramammary Paget's disease	✔	✔	✔	11/11
Case 10	Leiomyosarcoma	✔	✔	×	2/11
Case 11	Epidermoid cyst	×	✔	✔	5/11
Case 12	Cutaneous metastasis of lung SCC	✔	✔	✔	9/11
Case 13	Epithelioid sarcoma	✔	✔	×	3/11
Case 14	Cutaneous calcinosis	×	✔	✔	8/11
Case 15	Malignant peripheral nerve sheath tumor	✔	✔	✔	5/11
Case 16	Bullous pemphigoid	✔	✔	✔	11/11
Case 17	Herpes zoster	✔	✔	✔	10/11
Case 18	IgA vasculitis	✔	✔	✔	10/11
Case 19	Dermatomyositis	×	✔	✔	11/11
Case 20	Bazin's erythema induratum	✔	✔	×	9/11
Case 21	Plasma cell dyscrasia	×	×	×	1/11
Case 22	Buerger's disease	✔	✔	✔	5/11
Case 23	Necrotizing fasciitis	✔	✔	✔	8/11
Case 24	Tinea	✔	✔	✔	10/11
Case 25	Lichen sclerosis	×	×	×	1/11
Case 26	Pellagra	×	×	×	8/11
Case 27	Plasma cell cheilitis	✔	✔	✔	5/11
Case 28	Sweet's syndrome	✔	✔	×	7/11
Case 29	Junctional epidermolysis bullosa	✔	✔	✔	2/11
Case 30	Sarcoidosis	✔	×	✔	4/11

## Discussion

This study demonstrated that generative AI exhibited diagnostic performance comparable to dermatology specialists. Generative AI is particularly effective in diagnosing rare and complex diseases, and it is suggested that appropriate prompt engineering can significantly enhance its performance [8]. In this study, accuracy rates between AI models and specialists were correlated across different cases. However, there were instances where AI models made errors on cases that specialists accurately diagnosed, and vice versa, indicating potential differences in how AI and humans perceive diagnostic difficulty. Therefore, combining the strengths of both could improve diagnostic accuracy. In challenging cases, where specialists may struggle, AI-assisted diagnosis could enhance specialist performance, while human-guided prompt engineering could further refine AI accuracy. Generative AI has previously faced limitations in diagnostic accuracy due to constraints in generalizability, lack of evaluation metrics, and insufficient external validation across diverse datasets [9]. A meta-analysis of studies published between June 2018 and December 2023 on the diagnostic capabilities of generative AI models revealed significant variability depending on the model and medical specialty, with overall performance still lower than that of human physicians [10]. In the dermatological field, generative AI is similarly considered less accurate than board-certified dermatologists [5,6]. However, in this study, the latest generative AI models, ChatGPT-4o, Claude 3.5 Sonnet, and Gemini 1.5 Pro, demonstrated diagnostic performance comparable to that of dermatology specialists, suggesting rapid advancements in AI technology. It is anticipated that generative AI will become an essential diagnostic support tool in dermatology.

This study has several limitations. The sample size was small, with only 30 cases, which may have introduced selection bias. The number of specialists was also limited to 11, leading to variability in diagnostic performance. Additionally, both the specialists and the AI models might have been exposed to similar cases previously, potentially affecting accuracy rates. The presentation format, where all information was provided at once, differs from real clinical practice, limiting the applicability of the results. While generative AI is currently used mainly for information provision, it still has limitations in diagnosis and treatment. Future studies should evaluate AI's diagnostic capabilities in settings closer to real clinical practice, aiming for its integration as a complementary tool to specialist judgment.

## Conclusions

This study represents an initial evaluation comparing diagnostic accuracy between AI models and dermatologists. Future studies should expand the sample size and assess diagnostic performance under various conditions to further validate the practical applicability of AI as a supportive diagnostic tool. In conclusion, generative AI has the potential to supplement the variability in specialist diagnoses, thereby improving consistency and accuracy, suggesting its utility as an effective support tool in dermatological diagnosis. This study found that even among the generative AI models, ChatGPT-4o, Claude 3.5 Sonnet, and Gemini 1.5 Pro, response patterns differed. Combining diagnoses from multiple AI models may be important for enhancing diagnostic accuracy.

## Figures and Tables

**Figure 2:**
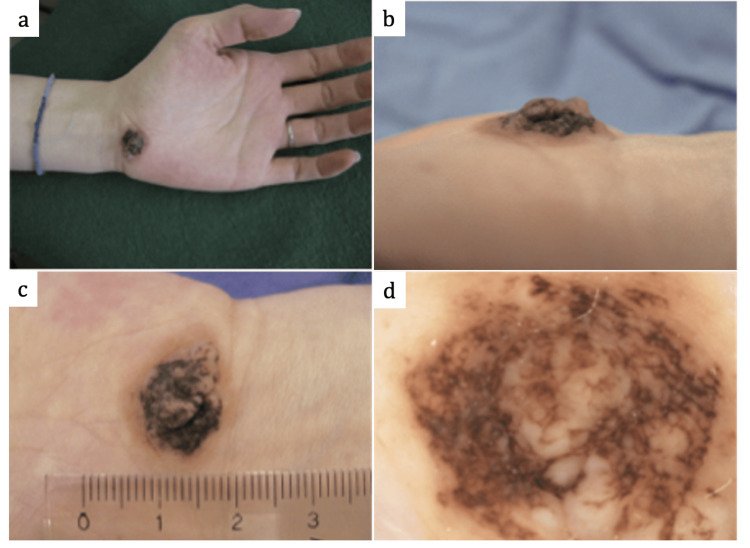
Clinical and dermoscopic findings of the lesion. (a) Clinical image showing the lesion on the left palm. (b) A closer view of the lesion, highlighting its elevated structure and black-brown appearance. (c) A lateral view of the lesion with size measurement. (d) Dermoscopic image displaying blue-white structures and an atypical lattice-like pattern without irregular streaks or a blue-whitish veil

**Figure 3:**
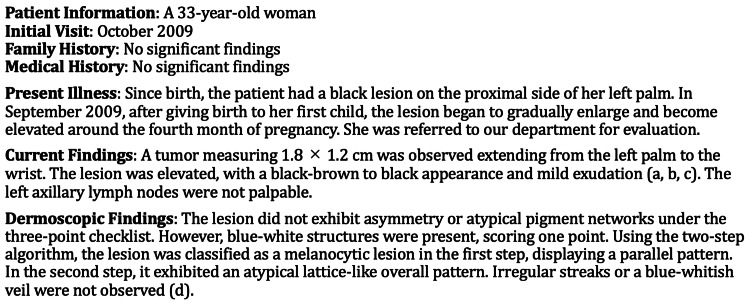
Clinical information of case 4, providing the basic information of the case, including patient demographics such as age and sex, chief complaints, present illness, and past medical history

**Figure 4:**
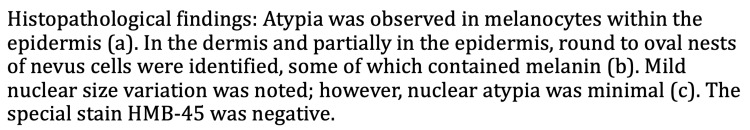
Clinical examination results of case 4, including laboratory data, imaging reports, and pathological findings

**Figure 5:**
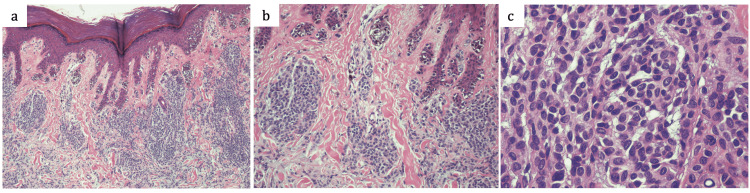
Histopathological findings. (a) Low magnification (H&E stain, 40×): atypia of melanocytes observed in the epidermal layer. (b) High magnification (H&E stain, 100×): increased numbers of basal melanocytes and circular-round nevus cells forming nests from the lower epidermal layer to the upper dermis. (c) High magnification (H&E stain, 400×): very low-grade nuclear atypia observed in nevus cells H&E: hematoxylin and eosin
